# Sex Differences in Temporal but Not Spatial Attentional Capture

**DOI:** 10.3389/fpsyg.2018.01893

**Published:** 2018-10-12

**Authors:** Tomoe Inukai, Jun I. Kawahara

**Affiliations:** ^1^Department of Psychology, Kobe Shinwa Women’s University, Kobe, Japan; ^2^Department of Psychology, Hokkaido University, Sapporo, Japan

**Keywords:** attentional capture, attentional control, sex differences, spatial visual search, temporal visual search

## Abstract

The accuracy of detecting or identifying a target decreases when a salient distractor is presented. This decrease is explained by the temporal or spatial diversion of attention to the distractor and thus is referred to as attentional capture. Using temporal and spatial visual search tasks, we examined whether there are sex differences in attentional capture. In Experiment 1A, a temporal visual search task measured attentional capture in the temporal domain by asking participants (97 men and 92 women) to identify a target embedded in a rapid stream of nontarget letters while ignoring a preceding peripheral distractor. In Experiment 2, a spatial visual search task measured attentional capture in the spatial domain by asking participants (146 men and 83 women) to detect a target among spatially distributed nontarget items while ignoring a distractor presented simultaneously. Our results indicate that attentional capture occurred in both tasks. In Experiment 1A, the magnitude of capture was significantly larger for women than men. In Experiment 1B, we confirmed sex differences in temporal attentional capture by recruiting a new set of participants (141 men and 85 women). In Experiment 2, the magnitude of capture was comparable between the sexes. These results suggest that women are more sensitive to bottom-up signals than men when they engage in a temporal search task and could be explained in terms of sex differences in the ability of adjusting the size of attentional window, within which attention is allocated to the most salient item.

## Introduction

Studies of cognitive function indicate that sex differences contribute to differences in cognitive performance among individuals. For example, there are sex-related differences in verbal and visuospatial tasks (e.g., Weiss et al., 2003; Rubia et al., 2010). Women are likely to outperform men on verbal fluency tasks, such as the Chicago Word Fluency Test, whereas men are likely to outperform women on navigation tasks (e.g., Sandstrom et al., 1998; Burton et al., 2005). Given that visuospatial information is tightly related to human behavior in selecting objects and in locomotion and spatial navigation, it is reasonable to assume that sex differences would also be found when participants selectively choose a target from nontargets in the spatial domain. Indeed, some studies (Brown, 2013) have demonstrated sex differences in attentional functions involved in spatial selection.

Specifically, an experimental study involving sex differences in selective attention demonstrated that a precue indicating a potential target location influences the allocation of attention. Precuing typically results in faster target detection at a cued location relative to uncued locations, even whe the cue is uninformative. Bayliss et al. (2005) reported that men consistently detect targets quickly regardless of the match between the cued location and the location where the target was presented, whereas women respond more slowly to a target presented at an uncued location than to a target presented at a cued location. Thus, they suggested that men allocate attention to specific locations while ignoring uninformative cues, whereas women do not show such a tendency.

However, it is notable that such sex differences could be attributable to women’s unsuccessful inhibition of uninformative cues rather than reflective attentional allocation to the cue. To examine this possibility, Stoet (2010) employed a flanker task to minimize shifts in attention and found that a preceding flanker stimulus located adjacent to a target caused a greater delay in detection for women than men when the flanker induced incompatible responses, unlike when it induced neutral responses. This finding suggests that women are more likely to fail to inhibit flanker response processes than men and are more susceptible to an incompatible flanker distraction.

By using a precuing paradigm designed by Posner et al. (1985), Brown (2013) found similar sex differences in inhibition ability. In that study, participants were required to detect a target appearing either to the right or left of the fixation point as quickly as possible. A cue indicating a potential target location appeared 1,400 ms before the target was presented. This long interval between cue and target produced an inhibition of return (IOR; e.g., Posner et al., 1985), in which reaction time to the target at a cued location was delayed relative to when the cue appeared at an uncued location. Importantly, the magnitude of IORs is larger for women than men. This finding suggests that women inhibit shifts in attention to an attended location more effectively than men.

This converging evidence of sex differences in selective attention suggests that women have a stronger attentional bias for new items and locations than men do. This leads to the possibility that the capability of controlling attention differs between the sexes. Previous studies have shown that attentional allocation occurs in three ways: by top-down control, by bottom-up control, and by reward history (e.g., Awh et al., 2012). Top-down control involves attentional allocation to an event or location to adaptively and flexibly accomplish a current behavioral goal using knowledge or by following instructions. Bottom-up control involves automatic and reflexive attentional allocation to a region containing salient events or oddball stimuli. These factors do not affect attentional allocation independently (e.g., Santangelo and Spence, 2008; Santangelo et al., 2011; Macaluso and Doricchi, 2013). For example, attention is automatically allocated to a specific location based on saliency and then reallocated to the next most salient location. At this time, top-down knowledge plays a role in determining whether attention is reallocated or not (e.g., Itti and Koch, 2000).

The selection of stimuli is also affected by a previous reward (Awh et al., 2012). An attentional bias for novelty specific to women (Stoet, 2010) suggests a sex difference in bottom-up attentional allocation when a salient but irrelevant stimulus produces bottom-up signals. That is, the ability to control attention could vary between the sexes when irrelevant but to-be-ignored objects exist. Thus, we examined this possibility in the present study using two types of cognitive behavioral tasks (spatial visual search and temporal visual search tasks) that were established to measure the effect of attentional control on attentional allocation in the presence of a distracting stimulus.

Some authors claim that participants fail to selectively search for a predefined target when a salient but task-irrelevant distractor is presented among the nontargets (e.g., Theeuwes, 1992; Folk et al., 2002). This failure is known as attentional capture and results in a decrease in performance in detecting or identifying targets in the presence of a salient, oddball distractor relative to a control condition in which no such distractors are presented. In one type of visual search task, the delay in reaction time is attributed to the diversion of spatial attention to the location of the singleton distractor followed by reallocation of attention to the target location (Theeuwes, 1992). In the other, it has been shown that attentional capture can also be found in temporal visual search tasks (Maki and Mebane, 2006). Folk et al. (2002) demonstrated that participants fail to identify a color-oddball target embedded among a central stream of nontargets in the presence of a peripheral oddball distractor compared to a control condition in which no peripheral distractor is presented. This observation suggests that attentional focus is diverted from the central location to the location of the peripheral distractor.

Based on the result that misallocation of attention to the distractor causes attentional capture during both spatial and temporal visual search tasks (e.g., Theeuwes and Burger, 1998; Leber and Egeth, 2006), these types of attentional capture obtained in the two different tasks (spatial and temporal capture) have been attributed to loss of top-down control in terms of diverting attention to a task-irrelevant distractor. Specifically, by manipulating the size of the attentional window, Theeuwes (2004) argued that attention is allocated to the most salient item within the window, regardless of the participants’ intentions. While the attentional window covers a whole search display during a spatial visual search (e.g., Belopolsky et al., 2007), it is gradually narrowed on a stream including a target during a temporal visual search (e.g., Jefferies and Di Lollo, 2009). This difference in the size of attentional window suggests the possibility that sex differences in attentional capture are modulated by the type of search task.

To examine whether the magnitude of spatial and temporal capture differs between the sexes, we tested participants in a spatial visual search and a temporal visual search task. In other words, we implemented these tasks to measure attentional control under the idea that a salient but task-irrelevant distractor should evoke a strong bottom-up signal that captures attention if participants fail to fully control allocation of attention. Moreover, if the cognitive mechanisms that are susceptible to the sex difference in inhibition reflect the ability to control bottom-up attention, a sex difference in attentional capture would be observed. Specifically, we assumed that attentional allocation might be more affected by distractors in women than men in a temporal domain but not in a spatial domain. This prediction is based on the suggestion that women would be unlikely to narrow the size of attentional window when they engage in a task because women failed to inhibit an item presented at next to a target compared to men (Stoet, 2010). If this were the case, the magnitude of temporal capture would be larger for women than men because women would be unlikely to narrow attentional window to focus a stream including a target flexibly, resulting in a presentation of distractor within the window. In contrast, the magnitude of spatial capture would be comparable between the sexes because women would not be required to adjust the size of attentional window during a spatial search task.

## Experiment 1A

In Experiment 1A, we examined whether there are sex differences in the magnitude of temporal capture during a temporal visual search task. To this end, participants were required to identify an oddly colored letter (i.e., the target) embedded among a rapid stream of nontarget letters while ignoring briefly preceding peripheral distractors. In this case, participants were forced to adopt a singleton detection mode, which is a strategy of searching for discontinuities, such as color and shape (Leber and Egeth, 2006). Temporal attentional capture is exemplified by an impairment in correct target identification when peripheral distractors appeared relative to when no such distractors were presented. If the ability to control temporal attention is comparable between the sexes, the magnitude of temporal capture would be unaffected by sex. Alternatively, if women are less likely to inhibit the distractors than men, because of their sensitivity to salient items, temporal capture would cause target identification to deteriorate in women more than in men when the distractors were presented.

### Materials and Methods

#### Participants

A total of 189 (97 men and 92 women) healthy undergraduate or graduate students from the National Institute of Advanced Industrial Science and Technology (AIST, Tsukuba, Japan) subject pool participated for pay. All reported normal or corrected-to normal visual acuity and normal color vision. This study was approved by the Institutional Review Board of AIST.

#### Apparatus and Stimuli

The stimuli were generated using MATLAB and Psychophysics Toolbox (Brainard, 1997; Pelli, 1997). They were displayed on a CRT monitor at a viewing distance of approximately 60 cm. Responses were collected via a keyboard connected to a computer.

A white plus sign was presented as a fixation point in the center of the screen. After the fixation point disappeared, 20 letters excluding I, O, Q, and Z, which were randomly chosen without replacement, were presented in brief succession at the same location. They subtended approximately 1.0° of visual angle in height and width. One was a uniquely colored (orange, blue, magenta, yellow, and red were used for half of participants, and orange, blue, magenta, yellow, and green for the other half) target letter. The nontarget letters were colored gray. The distractor consisted of four pound signs (i.e., #) at the same height and width as the letters, presented 5.2° above, below, to the right, and to the left of the letters. One of the pound signs was either red or green and the remaining items were gray. All stimuli were presented on a black background.

#### Procedure

**FIGURE 1:**
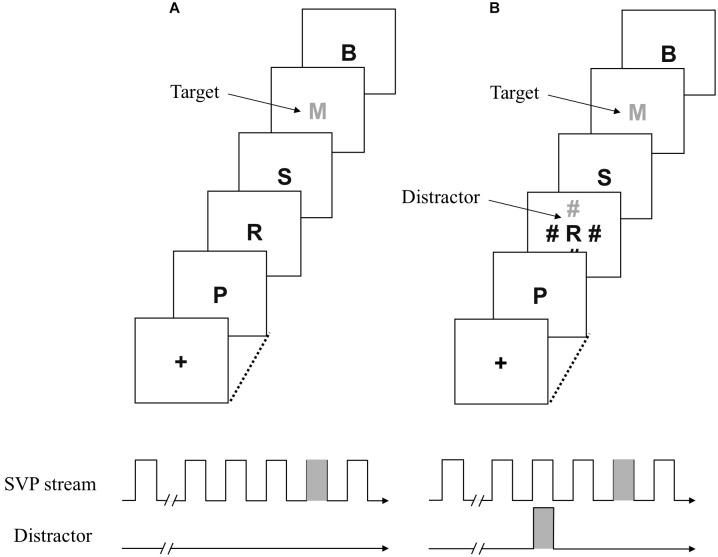
Schematic representation of events under the distractor-absent and distractor present-conditions (**A** and **B**, respectively) in Experiments 1A and 1B.

A fixation display was presented for 500 ms when participants pressed the space bar. The fixation cross was replaced with a central stimulus sequence consisting of 20 letters following a 500 ms blank interval. Each stimulus was presented for 43 ms, with an interstimulus interval of 43 ms, resulting in an 86 ms stimulus onset asynchrony. One of the central letters was presented as a target by displaying it in a target color (**Figure 1**). The target color was randomly chosen from the potential colors to force participants to adopt the singleton detection mode. The number of nontarget letters preceding the target varied randomly from 11 to 15. The distractors, if any, were presented 172 ms before the target. The location of a uniquely colored distractor (i.e., singleton distractor) was randomly chosen from the possible four positions. There were three conditions regarding the target–distractor relationship. Under the same-color condition, the target and distractor could be the same color. Under the different-color condition, the distractor was defined by a different color from the target. For a group of participants assigned to a target color list including red, the color of the distractor was red under the same-color condition, indicating that the distractor could be the same color with the target. The color of the distractor was green under the different-color condition, indicating that the distractor was always colored differently from the target. The distractor-absent condition was the control condition, in which the singleton distractor was not presented.

Participants identified the target letter while ignoring any distractors. After viewing all items in the central stream, participants pressed a key corresponding to the target and then hit the space bar to start the next trial. A low tone was sounded through headphones when an incorrect key was pressed. The same number of trials (60 trials) was assigned for these three conditions, which were administered in random order during the experimental session. Participants engage in 24 practice trials prior to the 180 experimental trials.

### Results

**FIGURE 2:**
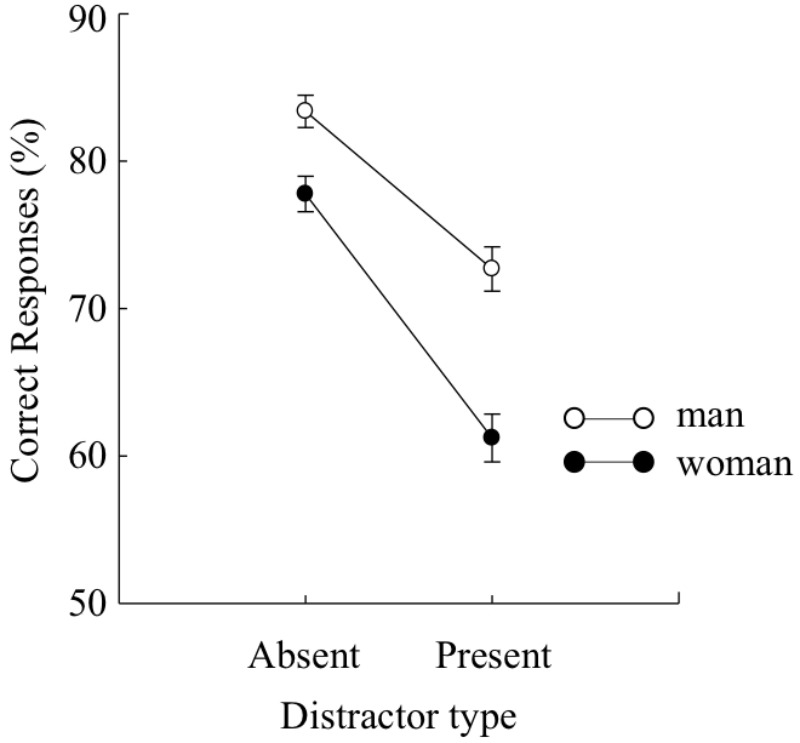
Mean percentages of correct target identification under each distractor type (distractor-present or distractor-absent) in Experiment 1A. Error bars indicate standard errors of the means.

The two participant groups assigned to target colors of either red or green were collapsed because no main effects or interactions were obtained in a preliminary analysis. We collapsed the type of distractor (same or different) for the analysis and compared accuracy under the distractor-absent condition as a general index of attentional capture, following the work of Inukai et al. (2010), which used the same procedure as the present study and found virtually identical identification accuracy of the same- and different-color conditions. The mean percentages of correct responses for each condition are presented in **Figure 2**. The mean percentages of correct responses for each condition were analyzed by a two-way mixed design ANOVA with sex (man or woman) as a between-participant factor and distractor type (distractor-absent or distractor-present) as a within-participant factor.

Additionally, we calculated the Bayes factor (BF) using JASP (JASP Team, 2018). BF is an indicator of which the alternative hypothesis is favored over the null hypothesis. There was a main effect of sex, *F*(1,187) = 25.17, *p* < 0.01, ${\text{η}}_{\text{p}}^{2}$ = 0.12, BF > 100, indicating that the accuracies of target identification were higher in men than in women. There was a main effect of distractor type, *F*(1,187) = 219.67, *p* < 0.01, ${\text{η}}_{\text{p}}^{2}$ = 0.54, BF > 100, indicating that the accuracies of target identification were higher under the distractor-absent condition than under the distractor-present condition. The interaction effect was significance, *F*(1,187) = 10.14, *p* < 0.01, ${\text{η}}_{\text{p}}^{2}$ = 0.05, BF = 15.52. A multiple comparison of the interaction indicated that the accuracies were significantly higher in men than in women, regardless of distractor type, *F*(1,374) = 8.42, *p* < 0.01, ${\text{η}}_{\text{p}}^{2}$ = 0.02 for the distractor-absent condition and *F*(1,374) = 35.13, *p* < 0.01, ${\text{η}}_{\text{p}}^{2}$ = 0.09 for the distractor present condition. As shown that the effect size of the distractor-present condition was larger than that of distractor-absent condition, importantly, the reduction in accuracies caused by the distractors was larger in women than in men.

### Discussion

This experiment was designed to examine the possibility of a sex difference in the ability to control attention. Given that women show greater sensitivity to new items and locations than men in cuing studies, the magnitude of attentional capture in women should be greater than that of men. The present study has two main findings. First, the accuracy of target identification was less when the distractors were presented than when no distractors were presented. This is a hallmark of attentional capture, suggesting that temporal attention was diverted to the location of the peripheral distractor. The result that temporal attention was captured regardless of the target and distractor colors confirmed that participants adopted the singleton detection mode to detect the target, consistent with previous findings that have demonstrated temporal capture.

Second, and importantly, the magnitude of temporal attentional capture differed between the sexes. The decreased accuracy of target identification under the distractor-present condition was more prominent in women than men, suggesting that women are more likely not to ignore the distractor and, thus, are likely to miss the target more often than men. That is, temporal attention in women is more susceptible to bottom-up control compared to men, resulting in deterioration of temporal capture. However, it remains unclear whether the susceptibility to bottom-up control in women is applied to spatial attention. If the result of this experiment could be caused by a possibility that women would be less likely to adjust the size of attentional window during a temporal search task (Jefferies and Di Lollo, 2009), women could expand the size of attentional window to encompass a whole search display before a spatial search task. If this were the case, the spatial capture effect could be comparable between the sexes. Before addressing this question, we replicated the effects of sex observed in this experiment with another set of participants in a similar temporal visual search task.

## Experiment 1B

### Materials and Methods

The apparatus, stimuli, and procedure were the same as those for Experiment 1A, with the following exceptions. A new set of 226 (141 men and 85 women) healthy undergraduate or graduate students from the AIST subject pool participated for pay. All reported normal or corrected-to-normal visual acuity and normal color vision. The target color was randomly chosen from among blue, yellow, white, red, or green. Under the same-color condition, a target color was always the same with the distractor. Under the different-color condition, the target color and the distractor color always differed. The color of distractor was randomly chosen from among the potential target color list. Each stimulus was presented for 50 ms, with an inter-stimulus interval of 50 ms, resulting in 100 ms stimulus onset asynchrony. A session consisted of 120 trials. Because Experiments 1A and 1B were originally designed for part of different projects, and color assignments were inconsistent across the experiments and irrelevant to the present purpose. Therefore, the detail of the factor of color assignment was not analyzed in the present study.

### Results and Discussion

**FIGURE 3:**
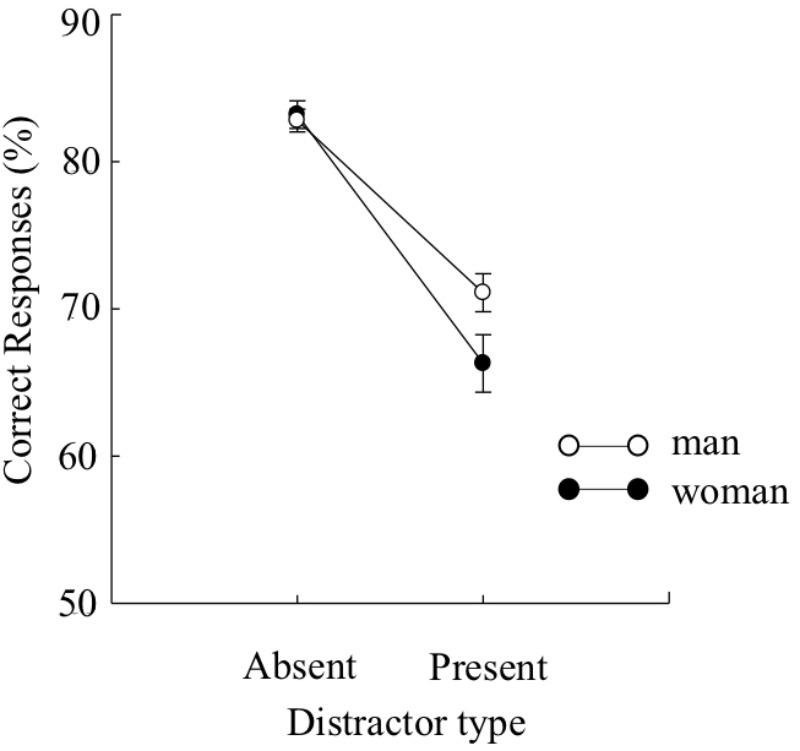
Mean percentages of correct target identification under each distractor type (distractor-present or distractor-absent) in Experiment 1B. Error bars indicate standard errors of the means.

The mean percentage of correct responses is plotted for each condition (distractor-present or -absent) in **Figure 3**. Because the Levene’s test (Levene, 1960) revealed that the variances for the distractor-present condition were not equivalent between the two groups, we arcsine transformed the data. A two-way mixed design ANOVA yielded a main effect of distractor type, *F*(1,224) = 193.39, *p* < 0.01, ${\text{η}}_{\text{p}}^{2}$ = 0.46, BF > 100, indicating that the accuracies of target identification were higher under the distractor-absent condition than under the distractor-present condition. There was no main effects of sex, *F*(1, 224) = 1.73, *p* > 0.1, BF = 0.26, indicating that the accuracies of target identification were comparable between the sexes. The interaction effect was significant, *F*(1,224) = 5.71, *p* < 0.05, ${\text{η}}_{\text{p}}^{2}$ = 0.02, BF = 2.15. Multiple comparisons run on the interaction indicated that the accuracies of target identification were significantly higher for men than for women when the distractor precedes the target, *F*(1,448) = 5.95, *p* < 0.05, ${\text{η}}_{\text{p}}^{2}$ = 0.01. However, the accuracies of target identification were comparable between the sexes when no distractors precedes the target, *F*(1,448) = 0.08, *p* > 0.1.

Temporal attention was diverted to peripheral distractors when participants adopted the singleton detection mode, resulting in less accuracy when identifying the target under the distractor-present condition than the distractor-absent condition. It should be noted that the magnitude of attentional capture was greater in women than in men. The present results were consistent with those in Experiment 1A in that the attentional bias for bottom-up signals is stronger in women than in men, in the presence of a task-irrelevant but salient item. Although we found sex differences in temporal capture in Experiments 1A and 1B, the performance in the distractor-absent condition differed in the two experiments. That is, a significant difference in this condition occurred between men and women in Experiment 1A but disappeared in Experiment 1B. The reason why this difference was obtained may be related to the differences in experimental design between these two experiments, such as the duration of target presentation.

## Experiment 2

The results of the preceding experiments indicated that the magnitude of temporal capture depended on the sex when in singleton detection mode, suggesting that women are more sensitive to bottom-up signals from peripheral distractors when they are engaged in the temporal visual search task. In the present experiment, we examined whether the same principle applies to a spatial visual search task. It is generally agreed that the two types of capture observed in temporal visual search and spatial visual search tasks can be attributed to an involuntary allocation of attention to distractors. If the result that the larger capture effect of Experiments 1A and 1B was observed in women than men would be caused by sex differences in the ability of adjusting the size of attentional window during a search task, the effects of sex observed in the previous experiments would be undetectable in a spatial visual search task. To determine this, we employed a task in which all stimuli, including a target and distractor, were distributed over space and were presented simultaneously. Participants discriminated the orientation of a line segment within a target shape embedded among nontargets while ignoring any color singleton distractor that appeared in the same search display (additional singleton task; Theeuwes, 1992).

### Materials and Methods

#### Participants

A new group of 229 (146 men and 83 women) healthy undergraduate and graduate students from the AIST subject pool participated for pay. All reported normal or corrected-to-normal visual acuity and normal color vision.

#### Apparatus and Stimuli

The apparatus was the same as that used in the previous experiments. The stimuli consisted of five, seven, or nine items equally spaced around a fixation point (white circle = 0.2° radius) on an imaginary circle (3.4° radius). Each stimulus consisted of a gray line segment (0.5°) surrounded by a green-outlined diamond (1.1° on a side) or circle (1.4° radius). The circle was presented as a shape singleton (i.e., a target) among uniform shaped nontargets (diamonds). One of the diamonds was replaced with a red-outlined diamond as a color singleton distractor, when presented. The line segments inside the diamonds were tilted 22.5° from the vertical or horizontal. The line segment inside the circle was a vertical or a horizontal line.

#### Procedure and Design

**FIGURE 4:**
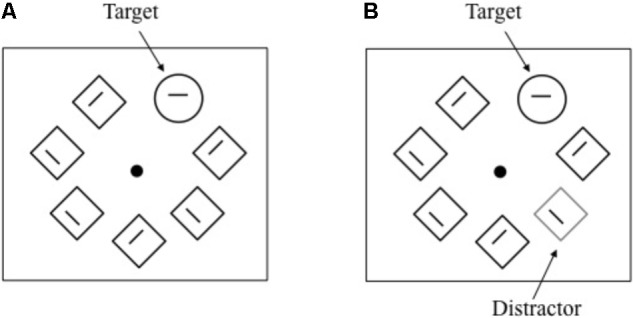
Schematic representation of events under the distractor-absent and distractor-present conditions (**A** and **B**, respectively) in Experiment 2.

Each trial started with a fixation dot, which expanded in size from a radius of 0.1° to 0.6° for 600 ms to warn the participant. Then, five, seven, or nine items were simultaneously presented around the fixation point, which shrunk 0.2° in radius. The number of nontargets was randomly determined across trials. In half of the trials, one of the nontargets was replaced with a singleton distractor (distractor-present condition), whereas no singleton distractor was presented (distractor-absent condition) in the remaining trials (**Figure 4**). The locations of the target and distractor were randomly determined.

The participants were required to identify the orientation of the line segment inside the target circle while ignoring the distractors by pressing one of two designated keys as accurately and quickly as possible. The factors of distractor type (present or absent) and set size (five, seven, or nine)^1^ were factorially combined, resulting in 240 experimental trials. The same number of trials (120) for the three set sizes and (120) for the distractor-absent conditions were administered in a random order during the experimental session. Participants received 15 practice trials prior to the experimental trials.

### Results

**FIGURE 5:**
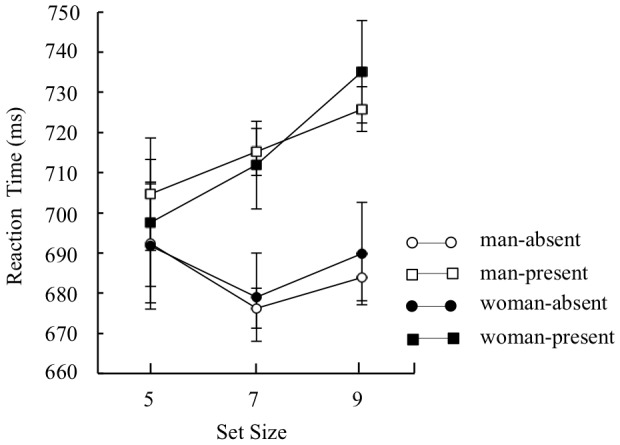
Mean reaction time of correct discrimination under each set size (5, 7, or 9) in Experiment 2. Error bars indicate standard errors of the means.

**FIGURE 6:**
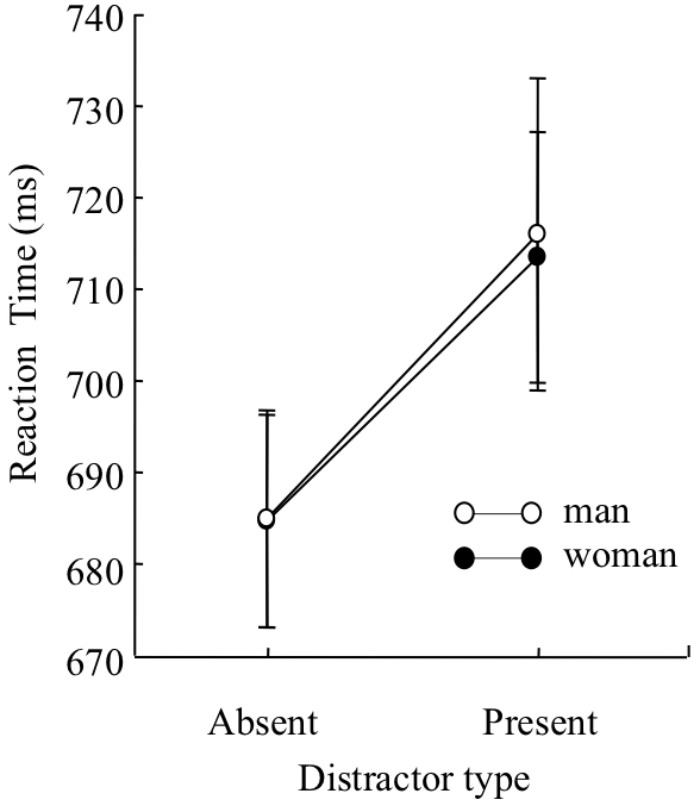
Mean reaction time of correct discrimination under each distractor type (distractor-present or distractor-absent) in Experiment 2. Error bars indicate standard errors of the means.

Mean target identification reaction times for each condition were analyzed by a three-way mixed design ANOVA with sex (man and woman) as a between participant factor, and distractor type (distractor-absent or present-present) and set size (5, 7, and 9) as within-participant factors (**Figure 5**). The analysis yielded a main effect of distractor type, *F*(1,227) = 50.84, *p* < 0.01, ${\text{η}}_{\text{p}}^{2}$ = 0.18, indicating that reaction time was faster under the distractor-absent condition than under the distractor-present condition. There was also a main effect of set size, *F*(2,454) = 5.31, *p* < 0.01, ${\text{η}}_{\text{p}}^{2}$ = 0.02, indicating that reaction time was longer on set size 9 than set sizes 5 and 7, *t*(454) = 2.81, *p* < 0.01 for set size 5, *t*(454) = 3.05, *p* < 0.01 for set size 7. A two-way interaction between distractor type and set size was significant, *F*(2,454) = 7.62, *p* < 0.01, ${\text{η}}_{\text{p}}^{2}$ = 0.03. Multiple comparisons run on the interaction yielded an effect of set size on the distractor-present condition, *F*(2,908) = 10.43, *p* < 0.01, ${\text{η}}_{\text{p}}^{2}$ = 0.02, indicating that reaction time was longer on set size 9 than set sizes 5 and 7, *t*(908) = 4.73, *p* < 0.01 for set size 5, *t*(908) = 2.72, *p* < 0.01 for set size 7. An effect of set size on the distractor-absent condition was marginally significant, *F*(2,908) = 2.59, *p* < 0.1, ${\text{η}}_{\text{p}}^{2}$ = 0.01. Because there were no sex-set size interactions, we collapsed the three set sizes (5, 7, or 9) to simplify the analysis, and compared mean target identification reaction times under the distractor-absent condition as a general index of attentional capture. Mean target identification reaction times for each condition are presented in **Figure 6**. Before conducting a two-way mixed design ANOVA with sex (man or woman) as a between-participant factor and distractor type (distractor-absent or distractor-present) as a within-participant factor, we confirmed the variances were equivalent between the two groups regardless of distractor type by Leven’s test. The analyses yielded a main effect of distractor type, *F*(1,227) = 51.66, *p* < 0.01, ${\text{η}}_{\text{p}}^{2}$ = 0.19, BF > 100, indicating that reaction time of target identification was significantly faster under the distractor-absent condition than under the distractor-present condition. However, a main effect of sex, *F*(1,227) = 0.003, *p* > 0.1, BF = 0.39, and interaction effect was not significant, *F*(1,227) = 0.20, *p* > 0.1, BF = 0.13. The ANOVA indicated that there were no differences in reaction time between sexes regardless of distractor type. Also, the BF supported the no significance of interaction effect.

### Discussion

The present experiments examined the sex difference in the ability to control attention during a spatial visual search task. The identification of a line segment in the target was delayed in the presence of a color singleton distractor compared to when no such distractor was present, implying attentional capture by the distractor. This result is consistent with the study of Theeuwes (1992). Importantly, the magnitude of the attentional capture was comparable between the sexes. Therefore, in conjunction with the results of the previous experiments, we suggest that the sex difference in attentional capture is limited to the temporal visual search task. These results would be attributed to sex differences in a flexibility in adjusting the size of attentional window.

Before proceeding to the General Discussion, it should be noted that the dependent measure differed across experiments in the present study (note 2). The main objective of the present study was to compare attentional capture effects during temporal and spatial search tasks. We chose the accuracy measure for the temporal capture task (Experiments 1A and 1B) and the reaction time measure for the spatial task (Experiment 2) because the vast majority of studies used these tasks for each domain. Reaction times have not been used frequently in temporal attentional capture studies because two separate responses are required for the two targets, although we used a single target-single response in spatial attentional capture task. Moreover, these two measures differ substantially in dynamic ranges and variances. Therefore, such a comparison may not help our arguments but introduce more concerns. To compare the two types of attentional capture, it would have been necessary to develop a new procedure to measure temporal attentional capture in reaction times or an equivalent to measure spatial attentional capture in accuracies. However, such an attempt was beyond our scope of the present study. Therefore, we chose the standard tasks to measure temporal and spatial tasks separately, to maintain procedural consistency across the previous and present study.

## General Discussion

In the present study, we examined the sex difference in attentional capture during temporal or spatial visual search tasks (**Table 1**). We predicted that attentional capture would be more exaggerated in women than men based on the findings that women are likely to direct attention to a new item (e.g., Stoet, 2010; Brown, 2013). Our results are consistent with our predictions, as a sex difference in attentional capture was observed during the temporal visual search task. In Experiments 1A and 1B, the magnitude of attentional capture differed between the sexes; the magnitude of attentional capture was greater for women than men. These results suggest that the control of temporal attention by women is more sensitive to bottom-up signals, such as abrupt onset and/or offset of visual objects outside the focus of the current participants’ goal, than is that of men. Importantly, this sex difference in the magnitude of attentional capture occurred only in the temporal domain, and it was not observed in spatial attentional capture (Experiment 2). We return to this issue later.

**Table 1:** Mean correct response rate with standard deviation and mean correct response time with standard deviation under the distractor-absent and distractor-present conditions in the temporal search and spatial search tasks.

Condition	Temporal search task (Experiment 1A)		Temporal search task (Experiment 1B)		Spatial search task						
	
	Correct response rate (%)		Correct response rate (%)		Response time (ms)							
	Man	Woman	Man	Woman	Man				Woman			
					5	7	9	All	5	7	9	All
Absent	83.38(10.72)	77.77(11.50)	82.79(9.21)	83.15(8.67)	692.40(178.92)	676.23(132.89)	683.95(157.56)	684.15(143.15)	691.81(144.16)	678.97(99.80)	689.88(116.33)	686.94(107.78)
Present	72.69(14.68)	61.22(15.42)	71.07(15.32)	66.27(18.00)	704.64(169.14)	715.20(156.95)	725.80(157.34)	715.32(155.32)	697.48(122.22)	711.89(124.77)	735.14(152.15)	714.44(124.37)

Superior attentional bias toward novelty found in women could be explained in terms of evolutionary theory, such as with a hunter–gathering theory, in which cognitive abilities differ between the sexes depending on their assigned roles (e.g., Eals and Silverman, 1994; Silverman et al., 2007). For example, women could have evolved to avoid an attended location and to direct attention to new locations for effective gathering as foragers, whereas men might have evolved to consider spatial information quickly and accurately for effective hunting. In such a theory, women would be always sensitive to bottom-up signals within a certain range because they tend to search for novel items and locations, resulting in increased attentional capture. Men would need to control their attention in a top-down way to grasp topography and follow prey. Therefore, they can ignore a task-irrelevant distractor during a temporal search better than women.

The results that the sex difference in attentional capture was limited to the temporal visual search task might reflect a flexibility in adjusting the size of attentional window. For example, in the study of Jefferies and Di Lollo (2009), participants were required to report two targets presented either on a same stream or different stream. As a result, they could report two targets presented on the different streams more correctly when a SOA between the two targets was short than when it was long. On the basis of this result, Jefferies and Di Lollo concluded that the size of attentional window is gradually narrowed on a stream including a target during a temporal visual search task. In a spatial search task, Belopolsky et al. (2007) found that the size of attentional window is set to cover a whole search display before starting to detect a target. If women are less likely to adjust the size of attentional window during a temporal search compared to men, it is reasonable that the sex difference in attentional capture would be limited to the temporal visual search.

Alternatively, the disengagement and reengagement processes of attention might differ between these two types of attentional capture. When attention is diverted to a peripheral distractor, the visual system reallocates attention to the location of a target for identification. Traditionally, it has been established that disengagement of attention from the peripheral distractor to a central target precedes reallocation of attention (Peterson and Posner, 2012). However, we assumed that the disengagement/reengagement processes can be disrupted by distractors during temporal search tasks. Inukai et al. (2010) provided evidence supporting this view. Specifically, they found less attentional capture when the duration between the onset and offset of the distractor was relatively long (approximately 200 ms) than when the duration was short (approximately 80 ms). Note that both distractors appeared at approximately 200 ms before a target presentation. They argued that when attention was diverted to the distractor, the diverted attention was recaptured by the same distractor when the offset of the distractor followed shortly after the onset. These results lead to the possibility that the sex difference that emerged in the temporal domain was due to the offset of the distractor immediately after its onset. Given that women are more sensitive to bottom-up signal changes in the temporal domain than men, it is reasonable to assume that greater capture effects occur for women than men.

## Ethics Statement

All experiments were approved by the Institutional Review Board of AIST, and written informed consent was obtained from all observers.

## Author Contributions

TI collected the data, wrote up the initial draft, and conducted the statistical analysis. JK designed the study, conducted the statistical analysis, and modified the draft.

## Conflict of Interest Statement

The authors declare that the research was conducted in the absence of any commercial or financial relationships that could be construed as a potential conflict of interest.

## Footnotes

1 The factor of set size, based on the literature, was originally introduced to examine whether attention was directed first to the distractor (Theeuwes, 1992). If the effect of set size is relatively small (i.e., shallow, approximately 10 ms/item, search slope as a function of set size) and the presence of the distractor delays the search, it is safe to argue that the search was efficient and, thus, attention was directed first to the distractor.
